# The FieldTrip-SimBio pipeline for EEG forward solutions

**DOI:** 10.1186/s12938-018-0463-y

**Published:** 2018-03-27

**Authors:** Johannes Vorwerk, Robert Oostenveld, Maria Carla Piastra, Lilla Magyari, Carsten H. Wolters

**Affiliations:** 1 Institute for Biomagnetism and Biosignalanalysis, University of Münster, Malmedyweg 15, 48149 Münster, Germany; 2 Scientific Computing & Imaging (SCI) Institute, University of Utah, 72 Central Campus Dr., Salt Lake City, 84112 USA; 3 Radboud University, Donders Institute for Brain, Cognition and Behaviour, Kapittelweg 29, 6525 EN Nijmegen, The Netherlands; 4 Department of Clinical Neuroscience, Karolinska Institutet, NatMEG, Nobels väg 9, 17177 Stockholm, Sweden; 5 Department of General Psychology, Faculty of Humanities and Social Sciences, Pazmany Peter Catholic University, Mikszath Kalman Square 1, Budapest, 1088 Hungary

**Keywords:** Source analysis, Forward modeling, Finite element method, Volume conductor modeling

## Abstract

**Background:**

Accurately solving the electroencephalography (EEG) forward problem is crucial for precise EEG source analysis. Previous studies have shown that the use of multicompartment head models in combination with the finite element method (FEM) can yield high accuracies both numerically and with regard to the geometrical approximation of the human head. However, the workload for the generation of multicompartment head models has often been too high and the use of publicly available FEM implementations too complicated for a wider application of FEM in research studies. In this paper, we present a MATLAB-based pipeline that aims to resolve this lack of easy-to-use integrated software solutions. The presented pipeline allows for the easy application of five-compartment head models with the FEM within the FieldTrip toolbox for EEG source analysis.

**Methods:**

The FEM from the SimBio toolbox, more specifically the St. Venant approach, was integrated into the FieldTrip toolbox. We give a short sketch of the implementation and its application, and we perform a source localization of somatosensory evoked potentials (SEPs) using this pipeline. We then evaluate the accuracy that can be achieved using the automatically generated five-compartment hexahedral head model [skin, skull, cerebrospinal fluid (CSF), gray matter, white matter] in comparison to a highly accurate tetrahedral head model that was generated on the basis of a semiautomatic segmentation with very careful and time-consuming manual corrections.

**Results:**

The source analysis of the SEP data correctly localizes the P20 component and achieves a high goodness of fit. The subsequent comparison to the highly detailed tetrahedral head model shows that the automatically generated five-compartment head model performs about as well as a highly detailed four-compartment head model (skin, skull, CSF, brain). This is a significant improvement in comparison to a three-compartment head model, which is frequently used in praxis, since the importance of modeling the CSF compartment has been shown in a variety of studies.

**Conclusion:**

The presented pipeline facilitates the use of five-compartment head models with the FEM for EEG source analysis. The accuracy with which the EEG forward problem can thereby be solved is increased compared to the commonly used three-compartment head models, and more reliable EEG source reconstruction results can be obtained.

**Electronic supplementary material:**

The online version of this article (10.1186/s12938-018-0463-y) contains supplementary material, which is available to authorized users.

## Background

In many applications of electroencephalography (EEG), it is desirable to reconstruct the active brain areas that generate the measured signals to achieve a better understanding of the neural processes. The reconstruction of these sources is called EEG source analysis; this reconstruction can be split into two mathematical problems, the EEG forward and the EEG inverse problem. Whereas the EEG forward problem consists of simulating the electric potential at the head surface that is generated by a microscopic source of brain activity, the EEG inverse problem aims at reconstructing a distribution of such sources that can explain the measured signal. Therefore, the accuracy of EEG source analysis directly depends on the accuracy that is achieved in solving the EEG forward problem.

The EEG forward problem in its quasi-static approximation is given by a Poisson equation with homogeneous Neumann boundary conditions

1 $$\begin{aligned} \nabla \cdot ( \sigma \nabla u )&= \mathbf {j}^p \quad \text { in } \Omega, \\ \langle  {\nabla u} , {\mathbf {n}} \rangle&= 0 \quad \text { on } \partial \Omega .\end{aligned}$$

*u* is the electric potential for which Eq. (1) is solved, $\sigma$ is the conductivity distribution in the head volume conductor $\Omega$, and $\mathbf {j}^p$ is the so-called primary current, i.e., a microscopic current source to model the brain activity, which is usually described by a current dipole $\mathbf {j}^p = \mathbf {m}\delta _{\mathbf {x}_0}$ with dipole moment $\mathbf {m}$ at position $\mathbf {x}_0$. A detailed derivation of the quasi-static approximation of the EEG forward problem can be found in [1, 2].

To solve the EEG forward problem with high accuracy, the volume conductor model $\Omega$ should reflect the head geometry as well as possible. The importance of detailed volume conductor models for an accurate inverse analysis has been demonstrated in various studies [3–5], especially the influence of distinguishing gray matter, white matter, and cerebrospinal fluid (CSF) instead of modeling a homogeneous brain compartment [6].

In order to be able to incorporate realistic head geometries $\Omega$, numerical methods to solve Eq. (1) are necessary. Different numerical methods have been proposed to solve the EEG forward problem (1), e.g., boundary element methods (BEM) [7–9], finite volume methods (FVM) [10], finite difference methods (FDM) [11, 12], or finite element methods (FEM) [13–17]. BEMs are commonly used in combination with simplified three-layer head models (skin, skull, brain), whereas FEM and FDM offer the possibility of modeling more complex geometries and also anisotropic conductivities, with only weak influence on the computational effort [6]. Finite element methods have been shown to achieve high numerical accuracies [13, 18], and the computational burden has been clearly reduced by the introduction of transfer matrices and fast solver techniques [19].

To solve (1) numerically, a discretization of the head domain $\Omega$ has to be generated. The FEM can be used with different kinds of head models. Surface-based tetrahedral head models generated from triangulations of the compartment boundaries allow for the accurate modeling of compartments of complicated shape, e.g., the strongly folded interface between cortex and CSF. These head models are generated based on surface triangulations of the compartment boundaries. Subsequently, a volume discretization of $\Omega$ into tetrahedral elements respecting these boundaries is generated using methods such as the constrained Delaunay tetrahedralization [20]. The surfaces have to be nonintersecting/touching and should have a sufficient distance between each other, which are constraints shared with the surfaces generated for BEM approaches. A common argument against the use of realistic surface-based tetrahedral head models that include more than the commonly used three compartments is the great effort that is necessary to construct these models.

The generation of the surface discretizations that are necessary for the construction of the tetrahedral head model can be especially complicated and time consuming. The additional consideration of skull holes—be it naturally existing ones such as the foramen magnum or those that are a consequence of brain surgery—as suggested by [12, 21], further complicates the generation of tetrahedral head models due to the more complicated compartment topologies. A possible approach to simplifying the head model generation is to use hexahedral head models generated directly out of segmented magnetic resonance images (MRIs) of the human head, which is done in this pipeline. To avoid the occurence of staircase effects, the generation of geometry-adapted meshes is implemented [22].

A further common argument against the wider use of FEM in praxis is the lack of easily accessible integrated software solutions. The goal of the pipeline presented in this paper is to resolve this problem. A MATLAB-based—and therefore multiplatform—FEM pipeline that is integrated in the FieldTrip-toolbox (http://www.fieldtriptoolbox.org, [23]) is presented and evaluated in this work. The pipeline allows for the easy computation of accurate solutions to the EEG forward problem using the FEM with automatically generated geometry-adapted hexahedral head models. Through the integration into FieldTrip, this pipeline also directly makes data preprocessing, as well as other tools for further analysis, e.g., source reconstruction, available. Furthermore, the integration into FieldTrip makes this pipeline available for users of other toolboxes such as EEGLAB (https://sccn.ucsd.edu/eeglab/) and SPM (http://www.fil.ion.ucl.ac.uk/spm/) that rely on FieldTrip for EEG forward computations.

In this manuscript, we describe the methodology we used to establish the pipeline, the implementation and workflow of the pipeline, a source reconstruction of somatosensory evoked potentials (SEP), and a basic evaluation of the accuracy of forward solutions computed with the obtained realistic five-compartment head model.

## Methods

### Segmentation and hexahedral mesh generation

As the first step to generate segmentations in the FieldTrip-SimBio pipeline, the SPM toolbox is used to compute masks of gray matter, white matter, and CSF based on a T1-MRI. A rough skull segmentation is created by dilating the union of these three masks, and a segmentation of the skin compartment is obtained by thresholding the MR image and subtracting the other masks.

Subsequently, a hexahedral mesh is generated directly based on this segmentation. To avoid staircase effects, geometry-adapted hexahedral meshes can be created in which mesh nodes at tissue boundaries are slightly shifted to obtain a more smooth representation of the boundaries [22, 24]. Examples of the use of geometry-adapted hexahedral meshes can be found in the studies of [25–27]; evaluations of the numerical accuracy achieved using geometry-adapted hexahedral meshes in sphere models were performed by [24, 28].

### The finite element method for solving the EEG forward problem

The presented pipeline employs a Lagrange (or continuous Galerkin) FEM approach, as it is commonly used for solving the EEG forward problem (1) using FEM [13–15]. In this approach, the potential *u* is approximated in the space of Lagrange functions $h_i (\mathbf {x})$. These functions are “hat functions” defined on the finite element mesh, i.e., they are piecewise linear and admit the value 1 on one node of the mesh and 0 on all other nodes. Inserting the $h_i$ into the weak form of Eq. (1) leads to the discrete system

2 $$\begin{aligned} Au = b. \end{aligned}$$

with

3 $$\begin{aligned} A_{ij}&= \int _\Omega \langle \sigma \nabla h_i , \nabla h_j \rangle dx, \end{aligned}$$

4 $$\begin{aligned} b_i&= \int _\Omega (\nabla \mathbf {j}^p) h_i dx. \end{aligned}$$

Solving Eq. (2) gives the discrete solution $u(\mathbf {x}) = \sum _i u_i h_i(\mathbf {x})$. For a more detailed derivation of the FEM, we refer to the standard literature, e.g., [29]. When making the common choice of $\mathbf {j}^p$ to be a current dipole, $\mathbf {j}^p = \mathbf {m}\delta _{\mathbf {x}_0}$, the right-hand side $b_i$ can no longer be evaluated directly, due to the singularity that is caused by applying the operator $\nabla$ to the $\delta$ function in $\mathbf {j}^p$. Multiple approaches have been developed to circumvent this problem. In our implementation, we apply the St. Venant approach, which approximates the current dipole through a configuration of current sinks and sources that evokes the same dipole moment. For a detailed description of the computation of the right-hand side vector $\mathbf {b} = \mathbf {b}^{ven}$ for the St. Venant approach and a comparison with other approaches for dipole modeling, we refer the reader to [24, 30, 31].

### Evaluation

Two kinds of evaluations are presented in this manuscript. To demonstrate the functionality of the pipeline, we performed a source reconstruction of SEP data using the FieldTrip-SimBio pipeline and visualized the results of the different computation steps. To offer a basic impression of the accuracy that can be achieved using the automatically generated five-compartment head models, we compared forward solutions obtained with such a five-compartment hexahedral head model generated using the FieldTrip-SimBio pipeline to forward solutions that were computed using highly detailed surface-based tetrahedral head models of the same subject that distinguished between three (skin, skull, brain) and six compartments (skin, skull spongiosa, skull compacta, CSF, gray matter, white matter).

#### Source localization of SEP data

We measured and evaluated a single-subject dataset consisting of MRIs and SEP data. All procedures were approved by the ethics committee of the University of Erlangen, Faculty of Medicine on 10. 05. 2011 (Ref. No. 4453). A healthy 23-year-old male volunteer subject was informed about the purpose of the study and gave written consent to participate, in accordance with local ethical regulations.

A T1-weighted (T1w-)MRI scan of the subject was acquired with a 3 T MR scanner (Magnetom Prisma, Siemens, Munich, Germany) using a 32-channel head coil. An MP-RAGE pulse sequence (TR/TE/TI/FA = 2300 ms/3.5 ms/1100 ms/8°, FOV = 256 ×  256  × 192 mm, voxel size = 1 × 1 × 1 mm) with water selective excitation was used. An 80-channel EEG and electrocardiography (ECG) were measured simultaneously. The EEG cap had 74 Ag/AgCl sintered ring electrodes placed equidistantly according to the 10–10 system (EASYCAP GmbH, Herrsching, Germany). In addition to the 74 electrodes, 6 channels were available and used for both eye movement detection (with a bipolar software montage) and source reconstruction. The electrode locations were digitized with a Polhemus Fastrak system (Polhemus Incorporated, Colchester, Vermont, USA) prior to the measurement. The EEG was measured with the subject in supine position to prevent erroneous CSF effects due to brain shift when combining EEG and MRI, following the results of [32]. To generate SEP data, one measurement run with electrical stimulation of the left median nerve and varying interstimulus interval (ISI) to avoid habituation (ISI: 350–450 ms, pulse duration 0.5 ms) was recorded at a frequency of 1200 Hz, resulting in 967 trials.

#### Head model accuracy

To evaluate the accuracy of the results achieved with the FieldTrip-SimBio pipeline, we compared forward solutions obtained with a five-compartment hexahedral head model generated using the pipeline to forward solutions that were computed using highly detailed surface-based tetrahedral head models of the same subject that distinguished between three (skin, skull, brain) and six compartments (skin, skull spongiosa, skull compacta, CSF, gray matter, white matter) and white matter anisotropy [6]. Otherwise, the computation pipeline to compute the forward solutions was not altered. The generation of the head models used in [6] involved extensive manual correction of the initial segmentation to obtain highly detailed surfaces of the compartment interfaces. This six-compartment (skin, skull compacta, skull spongiosa, CSF, gray matter, white matter) head model contains numerous details, such as realistic skull openings and white matter anisotropy. The simplified versions of the highly detailed tetrahedral head model were generated by neglecting some model details, as described below, to evaluate the effects of modeling or neglecting certain conductive compartments. A tetrahedral head model with a higher resolution was used as a reference to obtain the numerical error. In this study, we generated a five-compartment head model using the FieldTrip-SimBio pipeline within a few minutes, which is based on the same MRI data, and compared the accuracy of this simple model to that of the different versions of the tetrahedral head model.

The five-compartment hexahedral head model that was generated based on the segmentation of a T1-MRI using the FieldTrip-Simbio pipeline (Fig. 3) is denoted *5CI_hex_ft* (5 Compartment Isotropic HEXahedral FieldTrip) hereinafter. To classify the accuracy of the newly generated head model *5CI_hex_ft*, we compared it to different simplified head models as described in [6], starting from a three-compartment model (skin, skull, brain; *3CI*—3 Compartment Isotropic). Subsequently, a CSF compartment (*4CI*), gray and white matter distinction (*5CI*), skull spongiosa and compacta distinction (*6CI*), and white matter anisotropy (*6CA*—6 Compartment Anisotropic) were also modeled.

The electrode positions were aligned with the model surface. We regularly distributed source positions in the gray matter [6]; those that are valid positions in both the tetrahedral and the hexahedral head models (i.e., the mesh vertex next to the source position is fully inside the gray matter compartment) were selected, which led to 89,902 remaining sources. For each source position, a normal constraint was applied, i.e., the source direction was chosen to be orthogonal to the white matter surface. Reference solutions were computed using a high-resolution model *6CA_hr*.

As error measures, we used the relative difference measure (RDM), which is a normalized $\ell _2$-error that measures topography differences, and the logarithmic magnitude error (lnMAG), which measures magnitude differences to the reference solution [33, 34]:

5 $$\begin{aligned} \begin{array}{ll} RDM ( u^{num}, u^{ref} ) &{}= \left\| \frac{u^{num}}{\Vert u^{num} \Vert _2} - \frac{u^{ref}}{\Vert u^{ref} \Vert _2} \right\| _2 \\ lnMAG ( u^{num}, u^{ref} ) &{}= \ln \left( \frac{\Vert u^{num} \Vert _2}{\Vert u^{ref} \Vert _2}\right) \end{array} \end{aligned}$$

Here, $u^{num}$ is the test solution and $u^{ref}$ the reference solution. $\Vert \cdot \Vert _2$ denotes the (discrete) $\ell _2$-norm, i.e., $\Vert u \Vert _2 = \sqrt{\sum _i (u_i)^2}$. The minimal RDM value is 0 and the maximal error is 2; the lnMAG is centered around 0, and positive errors indicate an increased and negative errors a decreased magnitude compared to the reference solution.

## Implementation

The segmentation algorithm distinguishing the five compartments (white matter, gray matter, CSF, skull, skin) in the individual MRIs, as described in "Segmentation and hexahedral mesh generation" section, was already available in the FieldTrip toolbox (based on code of the SPM toolbox, http://www.fil.ion.ucl.ac.uk/spm/) through the function *ft_volumesegment*. Two additional features were required to enable the computation of EEG forward solutions using realistic multicompartment head volume conductor models: the generation of geometry-adapted hexahedral meshes from the segmented images and the computation of FEM forward solutions using these meshes. To obtain these functionalities, the required low-level code was implemented and integrated into the high-level functions of the common FieldTrip workflow.

### Hexahedral mesh generation

**Fig. 1:**
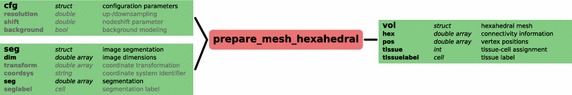
Sketch of the function *prepare_mesh_hexahedral*. Not all possible input parameters are shown. Optional parameters are indicated by gray font. Green background indicates MATLAB structs, red background MATLAB functions. Input variables are shown left, output variables right

For the generation of geometry-adapted hexahedral meshes, the function *prepare_mesh_hexahedral* was created; a sketch of the function call is shown in Fig. 1. This function allows the generation of geometry-adapted hexahedral meshes directly from segmented MR images. A basic five-compartment segmentation of a T1-MRI as input to this method can be generated using the function *ft_volumesegment* (cf. "Segmentation and hexahedral mesh generation" section). For more detailed (skull) segmentations, results from other toolboxes such as SPM (http://www.fil.ion.ucl.ac.uk/spm/), FSL (http://www.fmrib.ox.ac.uk/fsl), and BrainSuite (http://brainsuite.org) or from commercial tools like BESA (http://www.besa.de) and Curry (http://www.neuroscan.com) can be included at this point. Additional options for the mesh creation are generating geometry-adapted meshes with varying node-shift parameters (cf. "Segmentation and hexahedral mesh generation" section; [22, 24]), up-/downsampling of the image resolution, or modeling/not modeling the image background. It should be noted that unlike implementing the generation of hexahedral meshes and the fully MATLAB-based computation of FEM forward solutions on multiple platforms, improving the segmentation algorithm was not a main goal of the work presented here.

### EEG forward solution computation

**Fig. 2:**
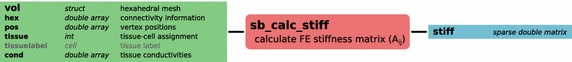
Sketch of the function *sb_calc_stiff*. Not all possible input parameters are shown. Optional parameters are indicated by gray font. Green background indicates MATLAB structs, red background MATLAB functions, blue background matrices. Input variables are shown on left, output variables on right

Following the mesh generation, the next necessary step was to enable the computation of FEM solutions for the EEG forward problem using a fully MATLAB-based multiplatform pipeline. Therefore, it was necessary to be able to calculate the stiffness matrix *A* (cf. Eq. (3), "The finite element method for solving the EEG forward problem" section). The approach we employed was to make the isoparametric FEM implementation from the SimBio toolbox (https://www.mrt.uni-jena.de/simbio/, [24]) directly accessible in MATLAB. A *MATLAB Executable* (MEX function) was implemented that enables the execution of the core *Fortran* functions of the SimBio toolbox from within MATLAB. The MEX function is implemented in Fortran and can be compiled on any platform for which a supported compiler is available (for supported compilers in MATLAB R2017b, see https://www.mathworks.com/support/compilers.html). The resulting function is *sb_calc_stiff*; a sketch of the function call is shown in Fig. 2. Pre-compiled binaries of this function for, e.g., most Linux distributions, macOS, and Windows 7/8/10, are available with the FieldTrip-toolbox.

All remaining code was directly implemented in the MATLAB programming language. The implemented functions include (in alphabetical order):

**sb_rhs_venant:** calculates the rhs-vector $b^{ven}$ (cf. (4); [24, 30, 31]); takes the mesh geometry and source position and direction as input; output is the rhs-vector $b^{ven}$;
**sb_set_bndcon:** sets the Dirichlet boundary conditions necessary to achieve a unique solution of Eq. (3); takes the stiffness matrix *A*, the rhs-vector *b*, the Dirichlet nodes, and the Dirichlet values as input; outputs are the stiffness matrix $\tilde{A}$ and rhs-vector $\tilde{b}$ with implemented Dirichlet boundary conditions;
**sb_solve:** solves the equation system (2) using a conjugate gradient solver with incomplete Cholesky preconditioning and zero fill-in (IC(0)-CG) [13]; takes the output from *sb_set_bndcon*, i.e., the stiffness matrix $\tilde{A}$ and rhs-vector $\tilde{b}$, as input; output is the solution vector *u*;
**sb_transfer:** computes the EEG transfer matrix $T^{eeg}$ [19]; takes the stiffness matrix, the mesh geometry, and the sensor positions as input; output is the transfer matrix.

**Fig. 3:**
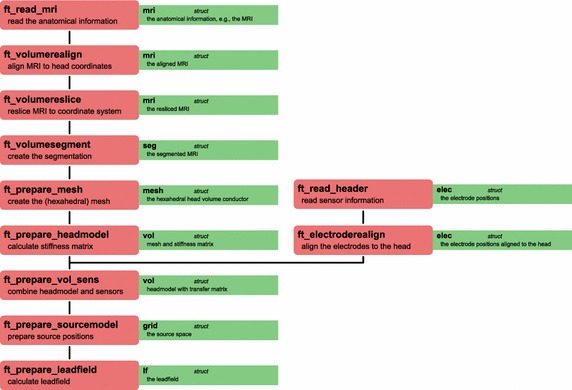
Sketch of the FieldTrip-SimBio pipeline (workflow goes from top to bottom). Red background indicates MATLAB/FieldTrip functions, green background (main) output of respective function

These low-level functions were integrated into the high-level functions of the FieldTrip toolbox to create an easy-to-use pipeline for FEM-based EEG forward simulations. The resulting pipeline is sketched in Fig. 3. Due to the FieldTrip workflow—which was originally designed for forward analysis using BEM or analytic spherical models—the main computational effort, i.e., the setup of the transfer matrix, is not included in the function *ft_prepare_headmodel* as one might expect from Fig. 3; instead, only the stiffness matrix *A* is computed in this function. The transfer matrix $T^{eeg}$ is subsequently computed in the function *ft_prepare_vol_sens*, where the sensor information is available to the pipeline functions for the first time (cf. Fig. 3).

## Results

### Source localization of SEP data

**Fig. 4:**
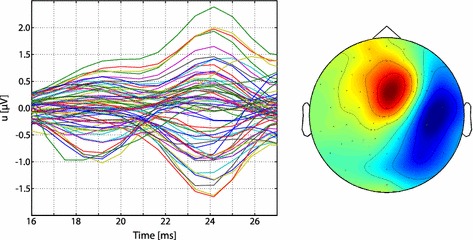
Butterfly plot of preprocessed SEP data (+16 to +27 ms, left) and peak topography (24 ms, right)

The EEG data were preprocessed using the FieldTrip functions *ft_definetrial*, *ft_preprocessing*, *ft_rejectvisual*, and *ft_timelockanalysis* (cf. *fieldtrip_simbio.m* in the Additional file 1). We applied a 20 Hz high pass filter, a 250 Hz low pass filter, and a discrete Fourier transform (DFT) filter for line noise removal at frequencies of 50, 100, and 150 Hz using *ft_preprocessing* [35]. A baseline correction was performed using the window from 150 to 50 ms before stimulus onset. The *ft_rejectvisual* function was used to reject bad channels and artifacts, e.g., due to eye-blinks. In total, 10 channels (C4, Pz, FC2, CP2, F1, C2, P6, AF8, TP8, PO7) and 104 trials were rejected, but we kept the additional channel LO2 because it was relatively free of artifacts, thus resulting in 65 channels available for source reconstruction and 863 trials for signal averaging. Finally, a time-locked average of the trials was computed with *ft_timelockanalysis*. A butterfly plot and the peak topography of the resulting data are shown in Fig. 4. The preprocessed SEP data can be downloaded from the Additional file 2 (*tlaLeft.mat*), and an introduction to data preprocessing using FieldTrip can be found on http://www.fieldtriptoolbox.org/tutorial/introduction.

**Fig. 5:**
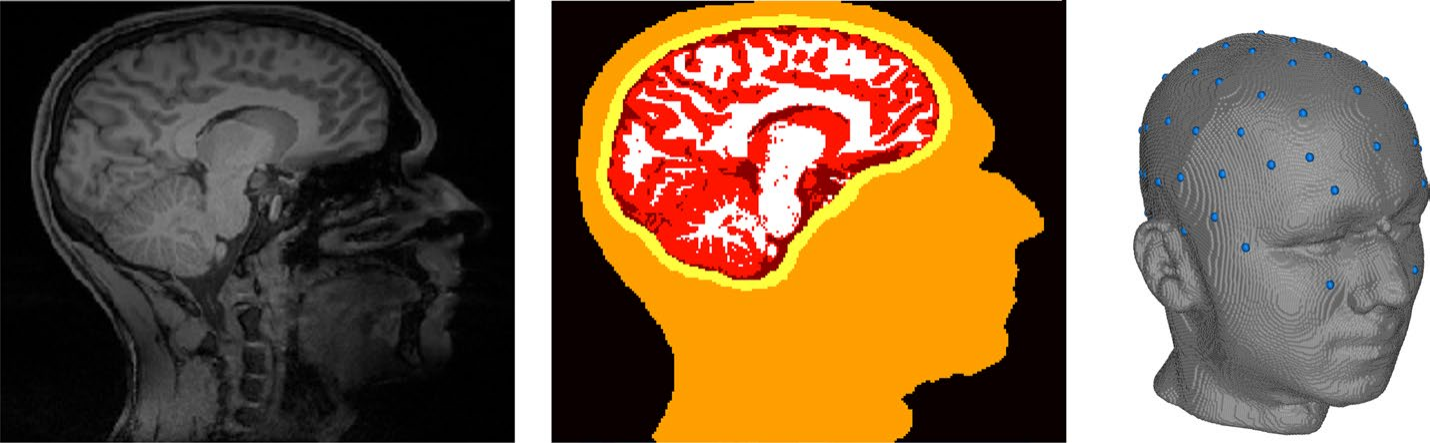
Original MRI (left), segmentation (middle), sagittal slice in T1-MRI space, and hexahedral mesh with aligned electrodes (right)

Following the pipeline sketched in Fig. 3, a hexahedral five-compartment head model was generated. A *scalpthreshold* of 0.06 was chosen instead of the standard value of 0.10 for *ft_volumesegment* and SPM12, which is the standard for brain segmentation in FieldTrip, was used, because it leads to a more accurate (at least visually) brain segmentation than SPM8. If necessary, the *brainthreshold* can also be adjusted to improve the quality of the brain mask, which was not necessary here. The resulting segmentation and the mesh with aligned electrodes are shown in Fig. 5. In the call of *ft_prepare_sourcemodel*, a grid resolution of 2 mm was chosen for the source space.

**Fig. 6:**
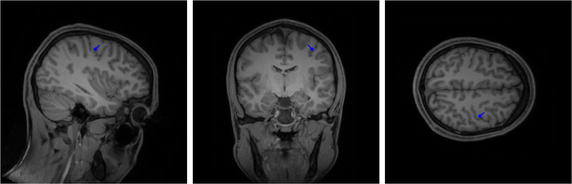
Result of source analysis of SEP data; sagittal (left), coronal (middle), and axial slice (right) in CTF-space, source visualized through blue arrow

Finally, the *P20/N20 SEP component* was localized at the peak (i.e., at +24 ms, cf. [36]) using the function *ft_dipolefitting*, which performs a goal function/dipole scan (when choosing the parameter *cfg.nonlinear = ‘no’*). The result of the source reconstruction is shown in Fig. 6; the *goodness of fit* (GoF) value was 0.963 (optimal value is 1). A sample script to perform the described steps can be found in the Additional file 2 (*fieldtrip_simbio.m*).

A complete execution of the P20/N20 source analysis, i.e., of the script *fieldtrip_simbio.m* (cf. Additional file 1), using a single core took about 7 h and 17 min on a PC running openSUSE Leap 42.3 with a 16-core Intel Xeon E5-1660 v3 CPU @ 3.00 GHz, 94 GB of DDR4-RAM, and a 476 GB SSD. The most time-consuming steps were the computation of the transfer matrix (*ft_prepare_vol_sens*) and the leadfield computation (*ft_prepare_leadfield*). The computation time can be reduced to below 1.5 h by running the computation of the transfer matrix in parallel on all 16 cores. Detailed computation times are listed in Table 1.

**Table 1:** Execution times of *fieldtrip_simbio.m* and the main executed FieldTrip functions (cf. Fig. 3, Additional file 1)

Step	Time [h:min:s]
Overall	7:17:10
ft_volumerealign, ft_volumereslice	0:00:01
ft_volumesegment	0:01:30
ft_prepare_mesh	0:00:22
ft_prepare_headmodel	0:03:11
ft_prepare_vol_sens	6:28:53
ft_prepare_sourcemodel	0:00:04
ft_prepare_leadfield	0:42:52
ft_dipolefitting	0:00:14

Computation performed single-threaded on a PC running openSUSE Leap 42.3 with a 16-core Intel Xeon E5-1660 v3 CPU @ 3.00 GHz, 94 GB of DDR4-RAM, and a 476 GB SSD

### Head model accuracy

**Fig. 7:**
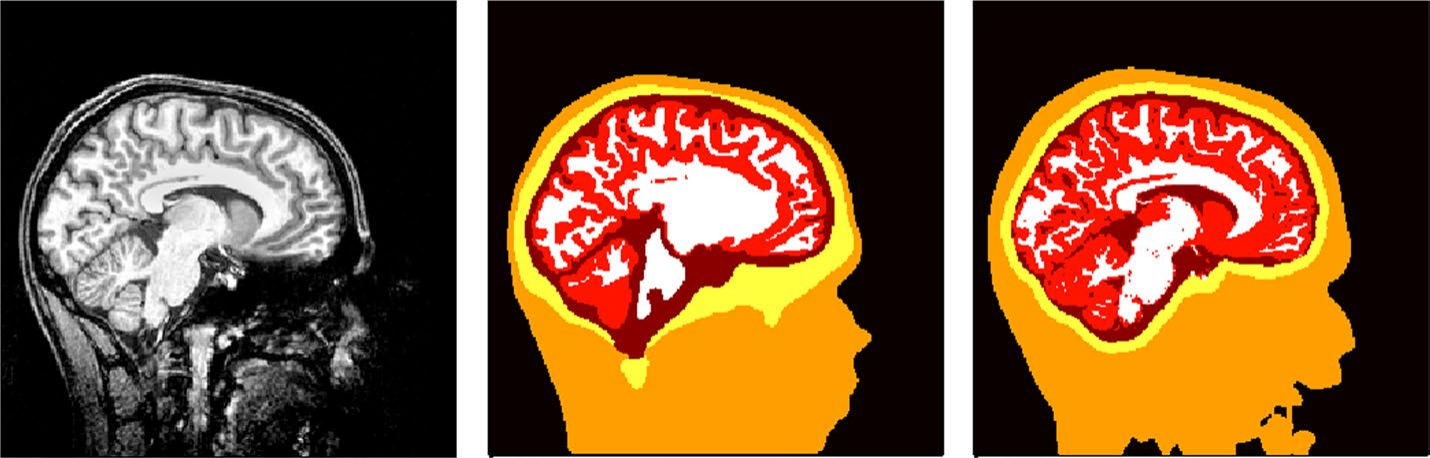
Original MRI (left), manually corrected segmentation (middle), and automatically generated segmentation using FieldTrip (right)

**Fig. 8:**
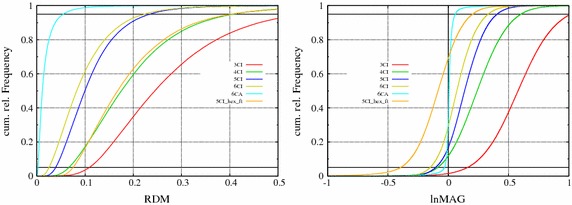
Cumulative relative frequencies of RDM (left) and lnMAG (right) of model simplification effects and error of model *5CI_hex_ft* with model *6CA_hr* as reference

We calculated the errors RDM and lnMAG in reference to a high-resolution model *6CA_hr* for all models and sources [6]. The segmentations used to create model *5CI_hex_ft* and models *3CI* - *6CA* are shown in Fig. 7. The resulting cumulative relative frequencies of the errors are shown in Fig. 8.

Comparing the fully automatic and the manually corrected segmentations (Fig. 7), it is clear that the main inaccuracies of the automatic segmentation are found for the skull mask, which is simply generated by a dilation of the inner skull surface in the FieldTrip pipeline, and in the nose/mouth area, where the contrast of the original image is low. The automatic segmentation of the brain compartments seems to be accurate, possibly even more accurate than the previously generated and manually corrected segmentation underlying the tetrahedral head model, where a minimal distance between outer brain and inner skull surface had to be assured to enable the tetrahedralization, and the ventricles were modeled as white matter to achieve a closed topology of the surfaces.

Figure 8 depicts the deviation of the forward solutions computed with model *5CI_hex_ft* in comparison to the modeling effects. At this point, only the errors of model *5CI_hex_ft* compared to the models *3CI*—*6CA* are discussed. For a detailed analysis of the differences between the models *3CI*—*6CA*, we refer the reader to the original publication [6]. With regard to the RDM, the errors are similar to those of model *4CI*, i.e., a highly detailed four-compartment model distinguishing skin, skull, CSF, and brain. Looking at the lnMAG, the results for the hexahedral model show a tendency toward an underestimation of source magnitudes. About 70% of the sources have a negative lnMAG value, and 90% of the lnMAG values are in the range from – 0.4 to 0.2. The error range is similar to model *5CI*.

## Discussion

In this paper, we presented and evaluated the FieldTrip-SimBio pipeline for finite element EEG forward computations in MATLAB. The pipeline was implemented to allow neuroscientists working with EEG to easily perform computations of EEG forward and inverse solutions using automatically generated five-compartment (skin, skull, CSF, gray matter, white matter) hexahedral head models and the finite element method. Our goal was to close the gap between methodological studies that show the high accuracy of the FEM and the practial challenges encountered by researchers in scientific praxis. We showed a source reconstruction of SEP data using this pipeline, and we evaluated the forward simulation accuracy that can be achieved with such a simplified head model in comparison to a highly detailed, manually corrected six-compartment tetrahedral head model for a test subject.

When comparing the simulation accuracy that was achieved with the head model generated using the FieldTrip-Simbio pipeline, *5CI_hex_ft*, with head models *3CI*—*6CA*, the five-compartment head model *5CI_hex_ft* performs about as well as the tetrahedral model *4CI* with regard to the RDM (Fig. 8). This result means that the RDM for model *5CI_hex_ft* is about the same as that of a highly detailed head model that includes the CSF compartment, but no distinction between gray and white matter, skull compacta and spongiosa, and also no anisotropic white matter conductivity (Fig. 8). With regard to the lnMAG, the absolute values of the error are of less interest, but a small spread of the errors to guarantee the comparability of the strength of different reconstructed sources is more important. Although the lnMAG values for model *5CI_hex_ft* are lower than for all other models in the comparison, the spread of the lnMAG is in the same range as that of model *5CI*. These results are remarkable given the negligible amount of time invested in model generation. As no manual corrections were applied for the segmentation, the pipeline presented here can be considered a button-press pipeline. The results show that through the distinction of CSF, gray matter, and white matter, accuracies that are at least comparable to model *4CI* are achieved, which is an important result given the influence of the highly conductive CSF compartment on the EEG forward solution [6]. Although only one test subject was considered here, the underlying segmentation algorithms have been evaluated in previous studies and shown to be accurate [37]. We therefore believe that these results offer the possibility to obtain an estimate of the expected accuracy of the EEG forward simulations calculated using the FieldTrip-SimBio pipeline in general.

In "Source localization of SEP data" section, a source analysis using measured SEP data (P20/N20 component) was performed. The results of the localization of SEP generated by medianus nerve stimulation are in line with the literature results (cf. Fig. 6; [36]). The overall computation time was about 7 h 17 min. The most time-consuming steps were the computation of the transfer matrix (in *ft_prepare_vol_sens*) and the leadfield matrix (*ft_prepare_leadfield*), with a time effort of about 6 h 29 min and 43 min, respectively. However, both steps can be easily parallelized within MATLAB with an optimal speed-up by using parallel loops (*parfor*). Several lines of the transfer matrix and several forward solutions can thereby be computed in parallel. For a fully parallel implementation, an overall computation time of less than one hour can already be achieved with an eight-core CPU, which can nowadays even be found in portable computers.

The main novelty that is presented in this paper is the possibility for researchers to easily use the St. Venant FEM approach for EEG forward computations from within the FieldTrip toolbox [35]. The St. Venant FEM approach was shown to achieve high numerical accuracies in a variety of studies, both in multicompartment sphere models, where an analytical solution exists and can be used as reference, and in realistic head models. The approach was also shown to be robust, e.g., achieving an accuracy that is essentially independent of the type of mesh, i.e., tetrahedral or hexahedral, the position of the source within the mesh, and the orientation of the source within the mesh, and to allow for fast computation times. The St. Venant FEM approach was compared to other FEM approaches, i.e., partial integration, subtraction, and Whitney, in multiple sphere model studies in both hexahedral and tetrahedral meshes and was shown to achieve the best combination of accuracy, robustness, and computation speed [13, 15, 31, 38]. Furthermore, the St. Venant FEM was also compared to two BEM approaches, the symmetric BEM as implemented in OpenMEEG [39] and a double-layer BEM approach, in both (tetrahedral) sphere models and in a realistic head model. Again, the St. Venant FEM was shown to achieve high accuracies and fast computation speeds [18]. This study also gave a first hint that differences in numerical accuracy between FEM and BEM approaches are often negligible compared to the effects of model simplifications, such as the use of three-compartment head models. Such head models are commonly used in combination with the BEM, which is the standard forward computation method in the FieldTrip toolbox. The effects of head model simplifications on EEG forward solutions in comparison to the numerical errors were later more thoroughly investigated [6], and a recommendation to distinguish at least five conductive compartments (skin, skull, CSF, gray matter, white matter) was formulated. Through the developments presented in this paper, it is now easy to address this recommendation using the FieldTrip toolbox. In "Head model accuracy" section, we demonstrated the improvements in forward simulation accuracy that can be achieved using a five-compartment head model generated with the FieldTrip-Simbio pipeline (head model *5CI_hex_ft*) in comparison to a three-compartment head model (head model *3CI*), which is commonly used in combination with BEM approaches. Given that the accuracy of the skull segmentation strongly differs in these two models, the improvements achieved by using a five-compartment head model over a three-compartment head model with the same skull segmentation are expected to be even greater and can be estimated by comparing the results for models *3CI* and *5CI*.

The main limitations of the presented pipeline concern the (skull) segmentation accuracy. As mentioned in the introduction, little work was invested in this study to improve the accuracy of the MRI segmentation. Differences between the automatically generated and the manually corrected segmentation were found for the segmentation of skull and brain compartment (cf. Figs. 5 and 7). The skull segmentation is generated by a dilation of the inner skull/outer brain surface in the FieldTrip-SimBio pipeline, which is a simple but robust approach. This segmentation results in a constant skull thickness and thereby a missestimation of the original skull thickness in many areas, which negatively affects the forward solution accuracy due to the major influence of an accurate modeling of the skull on EEG forward solutions [5, 12, 16, 17, 40]. The open nature of the pipeline presented here allows its users to include more accurate skull segmentations from other toolboxes such as SPM, FSL, or BrainSuite. A comparison study including these toolboxes was conducted in [37].

The restrictions of the tetrahedral mesh generation necessitate a sufficient distance between the inner skull and outer brain surface. This distance had to be artificially introduced and is a main cause for the visible differences in the brain segmentation. The significant effect of varying CSF thickness caused by movement of the brain with changing body position of the subject, as demonstrated by [32], may indicate that hexahedral meshes possibly allow for even more realistic modeling in this aspect as they facilitate realistically touching skull and brain compartments. The inaccurate segmentation of the nose/mouth area with FieldTrip should have only a minor influence because the model is nevertheless not cut off directly below the skull following the advice of [21]. The problem of accurately segmenting the scalp surface in the nose/mouth area occurred for only this single dataset, whereas the scalp surface could be nicely estimated using the FieldTrip-SimBio pipeline in "Source localization of SEP data" section (cf. Fig. 5). Thus, this erroneous segmentation is not a general problem of the segmentation algorithm, but occurs for only some MRI recordings.

Compared to the possible inaccuracies introduced through the limitations of the segmentation, the influence of numerical errors in the forward simulation is expected to be insignificant. As previously discussed, the St. Venant FEM approach achieves a high accuracy and is robust with regard to the possible influence of mesh type and structure. In general, so-called leakage effects, which occur when the thickness of the skull segmentation is only one layer of voxels, so that skull voxels are connected only via edges and nodes but not necessarily faces [41], are a possible source of error for the St. Venant FEM. However, in the presented pipeline, the thickness of the skull layer is ensured to be at least 3 mm, so that such effects would occur only at mesh resolutions of 4 mm or even coarser, which are not recommended due to the generally reduced simulation accuracy. The occurrence of leakage effects can be avoided for general head models with any skull thickness by the use of current-preserving FEM approaches, such as Mixed-FEM or discontinuous Galerkin (DG) FEM [16, 17]. A future development goal is to make the approaches implemented in duneuro (http://www.duneuro.org) directly accessible in FieldTrip.

Our results have shown that, using the easy-to-use and essentially automatic FieldTrip-SimBio pipeline, EEG forward solutions with accuracies that are comparable to those obtained with a manually corrected four- or five-compartment surface-based tetrahedral head model can be reached. Previously, the generation of such an accurate head model required a significant amount of nonautomatic model generation work. The pipeline thus offers a clear advantage when compared to the current standard of isotropic three-compartment head models that is still frequently used in EEG source analysis [39, 42, 43].

## Conclusion

This paper presented the FieldTrip-SimBio pipeline for the easy use of FEM-based EEG source analysis. Although the advantages of highly realistic multicompartment volume conductor models have been shown in multiple studies, the issue of the often high workload to create these models remained, especially for tetrahedral models. To allow the practical use of FEM approaches for EEG source analysis on several platforms, the FEM originally implemented in SimBio was integrated into a FieldTrip pipeline. We demonstrated that an automatically generated five-compartment head model achieved an accuracy that is clearly superior to that of the commonly used isotropic three-compartment head models. Furthermore, we demonstrated the analysis of SEP data using this pipeline, and obtained results that are in line with the literature.

## Additional files

**Additional file 1.** Sample matlab script *fieldtrip_simbio.m*. Example script for EEG source analysis using the FieldTrip-SimBio pipeline. Data preprocessing steps are included, but outcommented, use preprocessed data “tlaLeft.mat” from Additional file 2 instead.

**Additional file 2.** Example dataset: *tlaLeft.mat, mri.mat, segmentedmri.mat, elec_projected.mat*. Example dataset as processed in "Source localization of SEP data" section. It contains: *tlaLeft.mat*—Preprocessed SEP data (cf. Fig. 4); *mri.mat*—MRI of the subject (cf. Fig. 5, left); *segmentedmri.mat*—Segmented MRI (cf. Fig. 5, middle); *elec_projected.mat*—Electrodes aligned to the surface of the headmodel (cf. Fig. 5, right).
